# Managing the Heat Release of Calcium Sulfoaluminate Cement by Modifying the Ye’elimite Content

**DOI:** 10.3390/ma16062470

**Published:** 2023-03-20

**Authors:** Joelle Kleib, Georges Aouad, Mahfoud Benzerzour, Nor Edine Abriak, Mirvat Zakhour

**Affiliations:** 1 IMT Nord Europe, Institut Mines-Télécom, Centre for Materials and Processes, F-59000 Lille, France; 2 4515-LGCgE–Laboratoire de Génie Civil et géoEnvironnement, Institut Mines-Télécom, University Lille, University Artois, Junia, F-59000 Lille, France; 3 Laboratoire de Chimie Physique des Matériaux (LCPM/PR2N), EDST, Département de Chimie, Faculté des Sciences II, Université Libanaise, Fanar P.O. Box 90656, Lebanon

**Keywords:** calcium sulfoaluminate cements, heat of hydration, massive structure, mechanical properties

## Abstract

Nowadays, calcium sulfoaluminate cement (CSA) is garnering a large amount of attention worldwide and is being promoted as a sustainable alternative to Portland cement for specific applications. This study aimed to control the heat release of CSA cement paste by choosing the appropriate composition. For this purpose, different calcium sulfoaluminate clinkers with up to 75 wt. % of ye’elimite were synthetized. Then, a reactivity study on the synthesized clinkers was conducted while varying the amount of gypsum added. The heat of hydration was measured by isothermal calorimetry. The influence of the ye’elimite content on the heat release and on the compressive strength was investigated. According to the findings, the amount of ye’elimite in the cement has a direct relationship with the heat release. The heat release as well as the mechanical performance increase with the increase in the ye’elimite content in the CSA cement. An equation allowing the prediction of the total heat release after 24 h is provided. Such data can be of particular interest to consultants aiming at the reduction of thermal cracking in massive concrete.

## 1. Introduction

Calcium sulfoaluminate cement (CSA), considered as a green cement, is nowadays attracting more attention in the cement industry. This cement has already been used in China since 1970 in specific applications where rapid setting, early setting, and shrinkage compensation are required [1,2,3,4,5,6]. However, after the KYOTO conference, CSA has found its way to the European market. In fact, although ordinary Portland cement (OPC) is the most used construction material worldwide, its production accounts for approximately 5 to 8% of the man-made CO_2_ emissions [7,8,9,10,11]. The development of these special cements was prompted by the need to reduce CO_2_ emissions, energy use, and limestone usage [2,12,13,14,15,16,17,18]. The CSA clinker’s lower raw meal lime content (13–33% less than that of OPC cement), lower burning temperature (approximately 100–200 °C lower than that required for OPC manufacturing), and higher friability than the OPC clinker all contribute to its reduced carbon release [17,18,19,20,21,22]. In addition to its environmental benefits, CSA cement is commonly used for specific applications such as attaining high early and late strength and having a self-stressing material [14,16,17,23]. Recently, CSA cement has also been employed to reduce the alkali silica reaction [24], for waste stabilization [25,26,27], as an additive in the 3D printing of concrete [28,29], and to prepare low-carbon ecological ultra-high-performance concrete (UHPC) [30].

Ye’elimite C_4_A_3_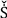 (conventional cement chemistry notation is used throughout this study: C = CaO; S = SiO_2_; A = Al_2_O_3_, F = Fe_2_O_3_, Ṧ = SO_3_; H = H_2_O) represents the main phase of the CSA clinker and its content can vary from 30 to 75% [31,32]. Other mineralogical phases can be found in the CSA clinker, such as belite, tricalcium aluminate, Al-rich ferrite, gehlenite, etc. [22,33,34,35].

Unlike OPC cement, whose mineralogy is clearly specified by normative aspects, there are currently no European or ASTM standards that regulate the composition of CSA cement [22].

However, the composition of the calcium sulfoaluminate cement (which includes the clinker’s composition as well as the amount of calcium sulfate added) is a critical parameter that controls its reactivity, mechanical performance, as well as its durability [36]. In this context, different studies have investigated the effect of sulfates on the heat of hydration released from CSA cement [15,37]. Chen et al. relate the hydration process to the ye’elimite and gypsum content of CSA cement [21,37]. The increase in the heat of hydration due to the early formation of ettringite–the main hydration product of ye’elimite–contributes to increasing the early compressive strength [38,39]. This important heat release could generate problems related to the shrinkage and cracking of massive concrete structures.

There are two ways to reduce the heat emissions in Portland cement. The first one is by substituting a part of the clinker with additions (such as pozzolan, fly ash, etc.), as described by the European standard NF EN 197–1 [40]. The second solution is by modifying the Portland clinker composition as described by the standards of ASTMC 150 [41] and leading to different types of cement. In fact, OPC cements with higher tricalcium silicate (C_3_S) and tricalcium aluminate (C_3_A) generate more heat and at a faster rate than other cements, as is the case of OPC cement type I according to the ASTMC 150 standard. Hence, for special applications wherein low heat release is required (in massive structures such as dams or mat foundations), as well as in hot weather, a type IV cement, which is a low-heat-of-hydration cement, was developed with a different mineralogical composition from type I cement. The average amounts of C_3_S and C_3_A dropped from 59 and 12%, respectively, in type I to 30 and 5%, respectively, in type IV cement. Consequently, the heat released at 7 days decreased from 350 kJ/kg in type I to 250 kJ/kg in type IV [41,42].

Regarding CSA cement, there are no published data related to the management of the heat release by altering the CSA clinker composition and the ye’elimite content in order to produce an equivalent to the type IV OPC cement.

Consequently, the main objective of this work is to control the heat release of CSA cement paste by modifying its composition. Different CSA cements with variable C_4_A_3_Ṧ content are produced at the laboratory scale and then the heat release is measured for each of the produced cements.

## 2. Materials and Methods

### 2.1. Materials

CSA clinkers were synthesized in the laboratory by mixing analytical-grade reagents: CaCO_3_, SiO_2_, Al_2_O_3_, and CaSO_4_.2H_2_O (gypsum). The raw mixes were prepared in order to reach the mineralogical compositions of the three CSA clinkers presented in Table 1. A backward calculation was performed to calculate the oxide composition and subsequently the weight ratio of the raw components in the raw meal in order to reach the mineralogical composition in Table 1. The difference between the three clinkers was the amount of ye’elimite (25, 50, and 75 wt. %). These percentages were chosen in order to cover the variability of the CSA composition within this range. Synthetized CSA clinkers are designated by the term “CSA”, followed by the percentage of ye’elimite. For example, the reference for a CSA clinker with 75 wt. % of ye’elimite is CSA75. The raw materials were mixed through a wet process in order to achieve better homogenization of the raw meal. After drying in an oven at 105 °C, the raw meal was pressed at 5 KN into pellets to obtain a more regular clinkering process. Pellets were placed in an alumina crucible and fired up to 1300 °C at a rate of 15 °C/min to 800 °C, and then at a rate of 8 °C/min to 1300 °C using a BLF Carbolite bottom-loading furnace. The clinker was cooled in the furnace after burning for 40 min at the clinkering temperature.

Cement was produced by mixing pure gypsum with the clinkers previously ground until a Blaine specific area between 3500 and 4000 cm^2^/g was achieved using a vibratory disc mill: RS 200 Retsch. Three rates of gypsum were used, corresponding to the molar ratios of sulfate/aluminate (SO_3_/Al_2_O_3_) equivalent to 0.5, 0.7, and 1. The corresponding CSA cements are referenced as those for the clinker, followed by the letter “G” and the value of the SO_3_/Al_2_O_3_ ratio (Table 2). Therefore, CSA75G0.5 represents CSA cement containing 75 wt. % ye’elimite and an amount of gypsum corresponding to a SO_3_/Al_2_O_3_ ratio of 0.5.

### 2.2. Methods

The mineralogy of the synthesized clinkers was studied using X-ray diffraction (XRD). A Bruker D2 with Cu Kα radiation was used. The X-ray patterns were acquired in the 2θ (10–80°) with a step of 0.02° and 1 s per step.

The reactivity of the cement was determined through isothermal calorimetric measurements performed at 20 °C. First, 6g of cement and 3.6g of water, previously stored at 20 °C, were mixed manually for a few seconds outside the calorimeter. Then, the mix was placed inside the measurement cells. The calorimeter used was a home-made calorimeter with fluxmeters that allowed the calorimeter to equilibrate in less than 5 min [26].

The compressive strength was determined on small cubes of 1 cm × 1 cm × 1 cm (due to the limited quantities of cement produced in the laboratory) made of cement pastes with a water/cement ratio (*w*/*c*) of 0.5. The filling of the molds was performed in two steps on a vibration table [31,43,44]. The samples were kept for 24 h in the molds, and then they were demolded and left to cure in a fully humid environment (RH = 100%, 20 °C) until the testing day. The compressive strength was measured on 6 samples after 1, 2, 7, and 28 days using an Instron 5500R-4206-006 press with a loading capacity of 1500 KN. The tests were performed at a constant displacement rate of 1.5 mm/min.

## 3. Results and Discussion

### 3.1. CSA Clinker Synthesis

**Figure 1:**
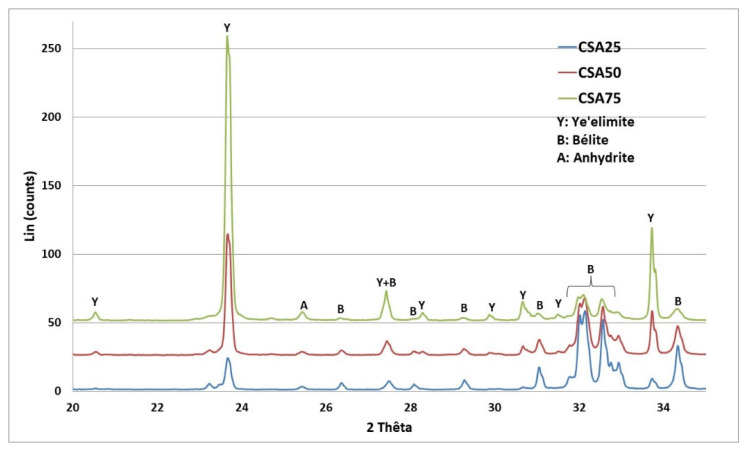
XRD pattern of the three CSA clinkers with different compositions.

The XRD analyses (Figure 1) conducted on the three clinkers synthesized with different ye’elimite content (25, 50, and 75 wt. %) show that the main crystalline phases are ye’elimite C_4_A_3_Š (Y), belite C_2_S (B), and anhydrite CŠ (A).

The increase in the ye’elimite content from 25 to 75% (CSA25 to CSA75) increases the intensity of the ye’elimite phases and decreases the belite ones. These findings demonstrate the effectiveness of the laboratory-based clinkering procedure.

After synthesizing the CSA clinkers, gypsum is added to the clinker and a study of the reactivity is performed on the cement pastes.

### 3.2. Effect of Gypsum Content on the Reactivity of CSA Cements

The quantity and reactivity of calcium sulfate added have a significant impact on the hydration of the CSA cement [20,34,45,46]. The quantity of the latter affects the nature and quantity of hydrates produced. Therefore, three rates of gypsum were chosen according to the equations of hydration (Equations (1)–(3)) and the sulfate/aluminate (SO_3_/Al_2_O_3_) ratio as follows:

C_4_A_3_Š + 18H → C_3_A.CŠ.H_12_ + 2AH_3_ (1)

2C_4_A_3_Š + 2CŠH_2_ + 52H → C_3_A.3CŠ.H_32_ + C_3_A.CŠ.H_12_ + 4AH_3_ (2)

C_4_A_3_Š + 2CŠH2 + 34H → C_3_A.3CŠ.H_32_ + 2AH_3_ (3)

Table 3 represents, for each of these equations, the corresponding SO_3_/Al_2_O_3_ ratio and the hydrates that are produced.

Accordingly, three different ratios were chosen 0.5, 0.7, and 1. These ratios consider the amount of sulfates coming from the CSA clinker.

Thus, SO_3_ = SO_3gypsum_ + SO_3clinker_ CSA.

Given that cement hydration is an exothermic process, the observation of the heat released can be correlated with the kinetics of the cement hydration. The reactivity of cement was thus followed by isothermal calorimetry at 20 °C.

**Figure 2:**
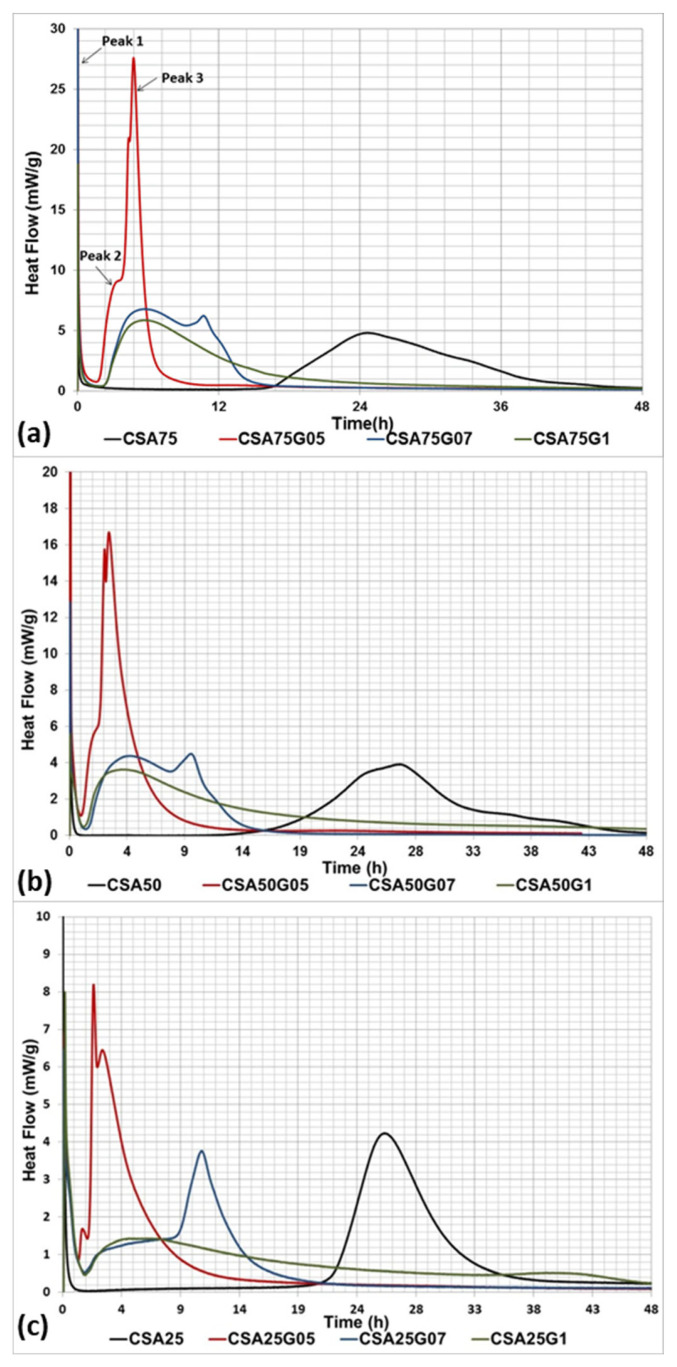
Heat hydration versus time of cement pastes containing (**a**) 75 wt. % ye’elimite, (**b**) 50 wt. % ye’elimite, and (**c**) 25 wt. % ye’elimite, with different SO_3_/Al_2_O_3_ ratios (0.5, 0.7, and 1 for CSA75G05, CSA75G07, and CSA75G1, respectively).

Figure 2 shows the calorimetric study for CSA cement with 75, 50, and 25 wt. % of ye’elimite, with and without adding gypsum, in order to study the effect of the gypsum content on the heat release of CSA cement. For the mix with 75 wt. % ye’elimite (Figure 2a), the clinker CSA75 (without gypsum) exhibits the first maximum (peak 1) shortly after water addition, which results from the very early hydration reactions and the dissolution of the anhydrous grains. Then, a long induction period of low thermal activity is observed for approximately 16 h. During this induction period, the mineralogical phases of the CSA clinker as well as gypsum dissolve slowly, and the coverage of the clinker grains by early hydration products occurs. Afterward, the heat flow increases and forms a second maximum after 24 h, where the main hydration reactions take place, leading to the hardening of the cement paste [47,48,49]. Adding gypsum, independently of its amount and of the SO_3_/Al_2_O_3_ ratio, decreases the duration of the induction period, leading to the acceleration of the CSA cement’s hydration. With the CSA–gypsum system, three peaks are identified. The first peak is common for all mixes and corresponds to the dissolution of the anhydrous grains. Then, an induction period of low thermal activity is observed. After this, the heat flow increases and forms a second peak (peak 2) at 3 h 30 min for CSA75G05 and at 6 h for CSA75G07 and CSA75G1. This second peak corresponds to further dissolution and the reaction between ye’elimite and gypsum to produce ettringite and aluminum hydroxide. The third peak (peak 3) is formed once the gypsum is depleted; therefore, the dissociation of ettringite will occur and the anhydrous grain will be available again for the hydration to produce monosulfoaluminate hydrate. This third peak occurs early in CSA75G05 (4 h 50 min), due to the small amount of gypsum added. In CSA75G1, the system is rich in gypsum; consequently, this peak does not appear during the study. Identical results were obtained by Winnefeld et al. when a CSA clinker (containing 64% of ye’elimite) was hydrated with different amounts of gypsum [50].

The results obtained with the mixes containing 50 and 25 wt. % ye’elimite (Figure 2b,c, respectively) are the same as those obtained with 75 wt. % ye’elimite. Therefore, independently of the amount of ye’elimite, for the same SO_3_/Al_2_O_3_ ratio, the hydration of CSA cement presents the same hydration mechanisms.

As a result, for the same composition, modifying the SO_3_/Al_2_O_3_ ratio modifies the heat release. For the rest of the study, one SO_3_/Al_2_O_3_ ratio is retained in order to be able to study the effect of the composition on the heat release and on the compressive strength. The literature shows that adding gypsum, according to the stoichiometry of reaction 3 (SO_3_/Al_2_O_3_ = 1), increases the risk of expansion [51]. Thus, 0.7 is chosen as the SO_3_/Al_2_O_3_ ratio.

**Figure 3:**
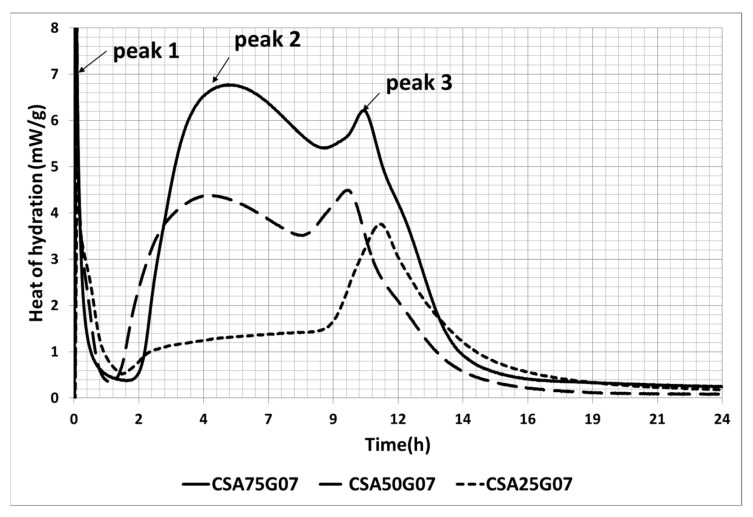
Heat of hydration versus time of the three CSA cement pastes with similar SO_3_/Al_2_O_3_ ratio (0.7).

Figure 3 shows the hydration of the three CSA cement pastes with the chosen SO_3_/Al_2_O_3_ ratio (0.7). It is clear that the heat flow increases with the amount of ye’elimite, so this latter represents the source of heat. Moreover, the third peak (corresponding to the depletion of gypsum) appears approximately at the same time for the three CSA cement pastes (between 10 and 11 h). This might be a result of considering the amount of sulfate coming from the CSA clinker in the SO_3_/Al_2_O_3_ ratio.

### 3.3. Heat Release Versus Ye’elimite Content

**Figure 4:**
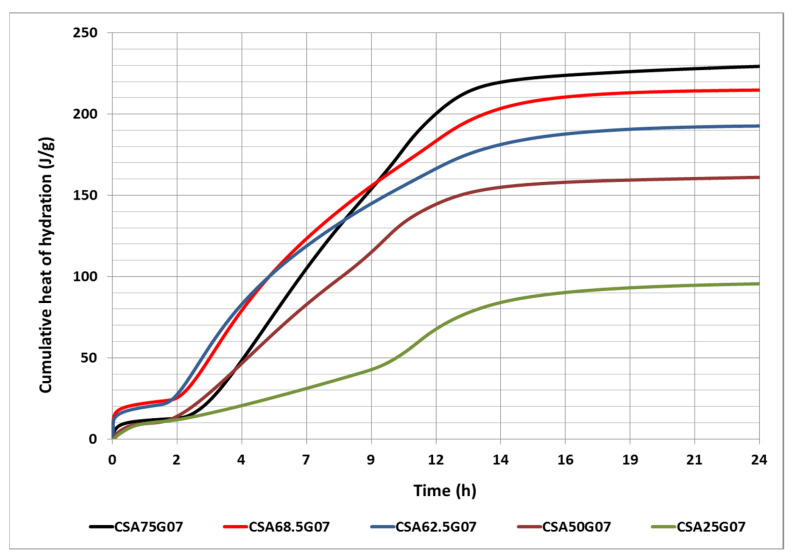
Evolution of the cumulative heat release for the different cement pastes in 24 h.

In this section, in order to better assess the effect of the CSA cement composition, and more specifically the effect of the ye’elimite content on the heat release of the cement paste, two additional clinkers were produced with 62.55 and 68.5 wt. % of ye’elimite. Figure 4 shows the cumulative heat release as a function of time for the five cement pastes. The findings demonstrate that as the ye’elimite content in the cement increases, so does the heat release. All cement pastes reach their maximum heat release after 24 h of hydration. This was supported by earlier research, which revealed that the calcium sulfoaluminate cement releases the majority of its hydration heat over the first 24 h [46].

**Figure 5:**
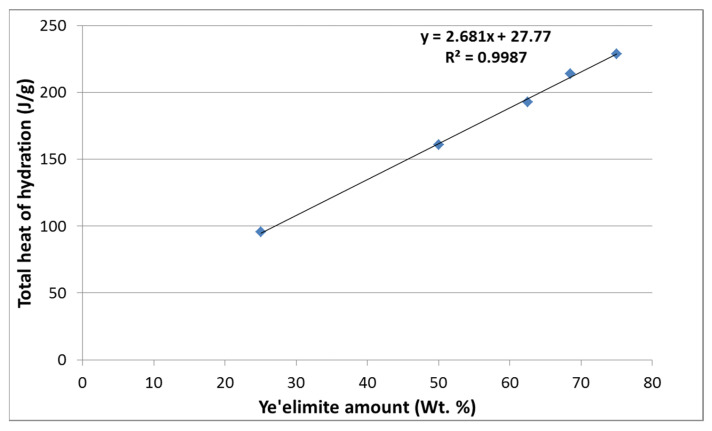
Total heat release at 24 h as a function of the ye’elimite content in the CSA clinker.

The evolution of the maximum heat release of cement pastes after 24 h of hydration, as a function of the ye’elimite content in the CSA cement, is represented in Figure 5. The results show that the heat release increases linearly with the ye’elimite amount in the clinker, with a high correlation factor (R^2^) of 0.998. This result offers a guideline to manage the CSA hydration heat release by modifying the ye’elimite content. Indeed, for an application with a maximum amount of heat release required, the maximum ye’elimite content can be calculated using the following Equation (4):

Total heat release after 24 h (J/g) = 2.68 x ye’elimite content wt. % + 27.77 (4)

### 3.4. Compressive Strength of Reference Cements

**Figure 6:**
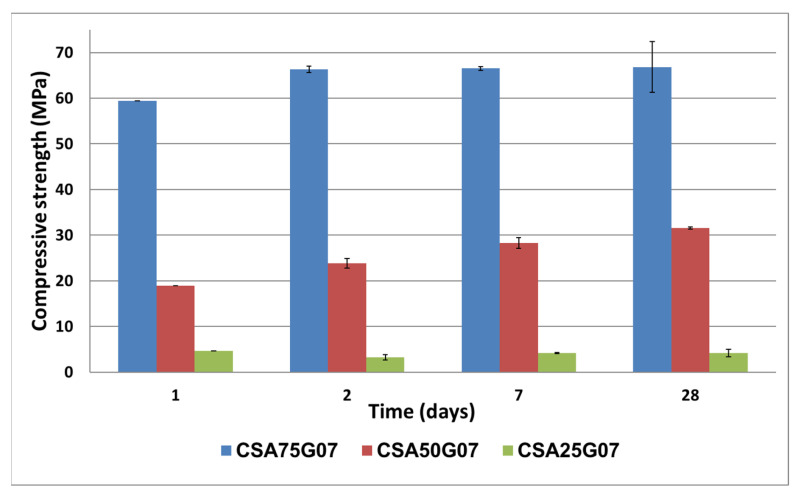
Compressive strength of cement pastes obtained after 1, 2, 7, and 28 days of curing.

A compressive strength test is conducted on the cement pastes at 1, 2, 7, and 28 days (Figure 6). The results show that the compressive strength increases with the amount of ye’elimite phase in the CSA clinker. This strength increases rapidly during the first two days and then slows down until it reaches a strength plateau of 66, 31, and 4.6 MPa for CSA75, CSA50, and CSA25, respectively.

This result could be due to the fact that the quantity of ettringite produced–which confers the mechanical properties–increases with the amount of ye’elimite, leading to an increase in the compressive strength of the different samples.

## 4. Discussion

The synthesized CSA cement with different amounts of ye’elimite content and an identical SO_3_/Al_2_O_3_ ratio showed identical mechanisms of hydration but a different amount of heat released, which increased with the increase in the ye’elimite content. The ye’elimite phase is the most reactive phase in the CSA cement, and the literature shows that the belite phase starts to react after several days of hydration [45]. Therefore, it can be considered that the main source of heat during the first 24 h of hydration is derived from the ye’elimite’s hydration. In addition, the results of the compressive strength test are consistent with the calorimetric study and show that the CSA cement pastes reach almost their maximum strength at 1 day. Consequently, the cumulative heat released after 24 h of hydration depends on the composition of the CSA clinker and more specifically on the ye’elimite amount. Therefore, regarding the objective of this paper, and how to manage the heat release of the CSA clinker by modifying its composition in order to produce an equivalent to type IV OPC cement, the results show that an equation with a direct relation between the ye’elimite content and the heat released could be applied. This equation depends only on the ye’elimite content because, in the short term, the belite phase does not react. Hence, knowing the heat released required for an application, the composition of CSA can be deduced. This work showed that, as for Portland cement, where the ASTMC 150 standard defines the composition of the different types of cement by modifying the phases’ (C_3_S, C_2_S, C_3_A, and C_4_AF) content, the same approach could be applied for CSA cement by modifying the C_4_A_3_Ṧ content.

## 5. Conclusions

The purpose of this work was to study the variability of calcium sulfoaluminate cement CSA and to control the heat release of CSA cement paste by modifying its composition.

From the above study, we can obtain the following conclusions.

- For the three synthesized CSA clinkers, the phases ye’elimite, belite, and anhydrite are obtained.
- The calorimetric measurements show that the gypsum quantity is a crucial parameter on which hydration depends.
- For the chosen ratio SO_3_/Al_2_O_3_ = 0.7, the calorimetric results show that gypsum is depleted approximately at the same time regardless of the amount of ye’elimite in the CSA clinker. This could be due to the consideration of the sulfate coming from CSA clinker in the calculation of the SO_3_/Al_2_O_3_ ratio.
- The heat release increases linearly with the ye’elimite content in the CSA clinker. An equation allowing the prediction of the total heat release after 24 h is set up.
- Guidelines to manage the CSA hydration heat release by modifying the ye’elimite content are developed. Accordingly, for an application where a specified heat release is required, an equivalent to type IV Portland cement could be produced for CSA cement by changing the ye’elimite ratio in the clinker composition, as is done for C_3_S and C_3_A in Portland cement.
- The compressive strength increases with the increase in the amount of ye’elimite in the CSA clinker.

## Figures and Tables

**Table 1:** Mineralogical composition of the three synthetized CSA clinkers (wt. %).

References	Mineral Composition		
	C_4_A_3_Š	C_2_S	CŠ
CSA75	75	23	2
CSA50	50	48	2
CSA25	25	73	2

**Table 2:** References of synthetized CSA cement with different SO_3/_Al_2_O_3_ ratios.

SO_3_/Al_2_O_3_	CSA Cement	SO_3_/Al_2_O_3_	CSA Cement	SO_3_/Al_2_O_3_	CSA Cement
0.5	CSA75G0.5	0.7	CSA75G0.7	1	CSA75G1
	CSA50G0.5		CSA50G0.7		CSA50G1
	CSA25G0.5		CSA25G0.7		CSA25G1

**Table 3:** Hydrates produced for each SO_3_/Al_2_O_3_ ratio.

Equation	SO_3_/Al_2_O_3_ Ratio	Hydrates
Equation (1)	1:3	AFm (monosulfate hydrate), amorphous aluminum hydroxide (AH_3_)
Equation (2)	2:3	AFt (ettringite), AFm (monosulfate hydrate), amorphous aluminum hydroxide (AH_3_)
Equation (3)	1:1	AFt (ettringite), amorphous aluminum hydroxide (AH_3_)

## Data Availability

The data presented in this study are available on request from the corresponding author.
